# Supplementary material for: Advances in Low-Density Flexible Polyurethane Foams by Optimized Incorporation of High Amount of Recycled Polyol
Source: Polymers (Basel). 2021 May 26;13(11):1736. doi: 10.3390/polym13111736 (PMC8198888; doi:10.3390/polym13111736)
Supplement: Supplementary file 1 [file polymers-13-01736-s001.zip › polymers-1204935-supplementary.pdf]

Advances in Low Density Flexible Polyurethane Foams by Optimized Incorporation of High Amount of Recycled Polyol

Gabriel Kiss [1,2], Gerlinde Rusu [1*], Geza Bandur [1], Iosif Hulka [3], Daniel Romecki [4], and Francisc Péter [1,3]

[1]University Politehnica Timișoara, Faculty of Industrial Chemistry and Environmental Engineering, C. Telbisz 6, 300001 Timișoara, Romania
[2]Momentive Performances Materials, Carl-Duisberg-Straße 101, 51373 Leverkusen, Germany
[3]University Politehnica Timișoara, Research Institute for Renewable Energies, G. Muzicescu 138, 300501, Timișoara, Romania
[4]Ikano Industry Sp. z o.o., Magazynowa 4, 64-610 Rogoźno, Poland
*Corresponding author: gerlinde.rusu@upt.ro

Supplementary material

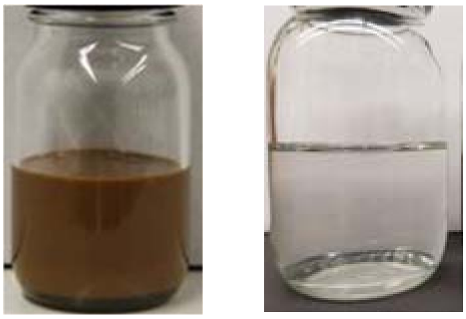

(a) (b)

Figure S1. Pictures of Repolyol (a) and reference polyol Voranol 3322 (b)

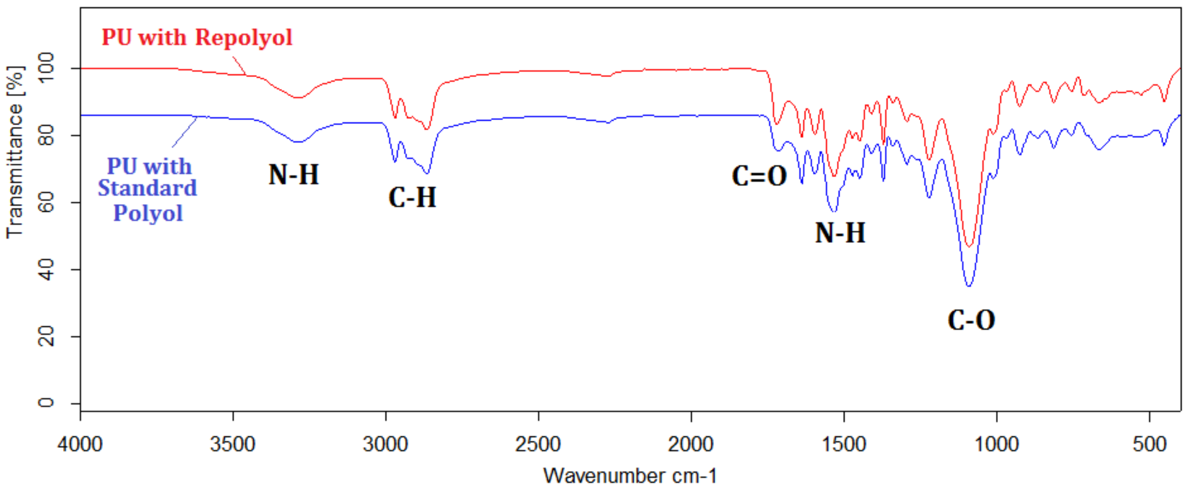

Figure S2. FTIR spectra of the polyurethane foams obtained with Repolyol (red line) and reference polyol (Voranol 3322, blue line)
